# Supplementary material for: The Incidence, Survival, and HPV Impact of Second Primary Cancer following Primary Oropharyngeal Squamous Cell Carcinoma: A 20-Year Retrospective and Population-Based Study
Source: Viruses. 2022 Dec 22;15(1):34. doi: 10.3390/v15010034 (PMC9867066; doi:10.3390/v15010034)
Supplement: Supplementary file 1 [file viruses-15-00034-s001.zip › Supplementary Material.pdf]

viruses

MDPI

Supplementary Materials. The following supporting information can be downloaded at: https://www.mdpi.com/article/10.3390/v15010034/s1, Figure S1: Model control testing for linearity of the continuous covariables by plotting the Martingale residuals against continuous covariables; Figure S2: Proportionality of the variables tested by log-minus-log curves showed in Schoenfeld residual plots.

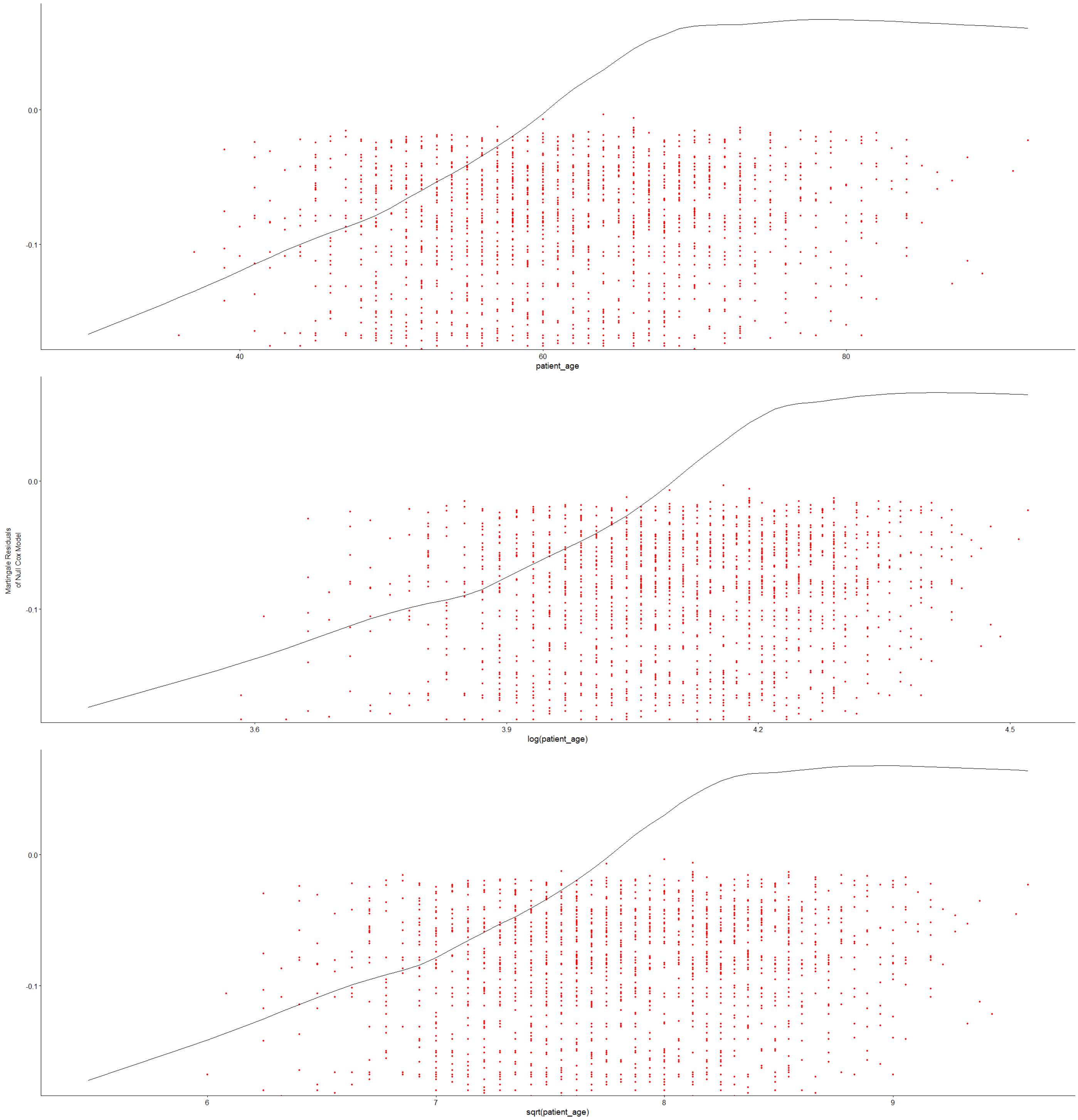

Figure S1. Model control testing for linearity of the continuous covariables by plotting the Martingale residuals against continuous covariables.

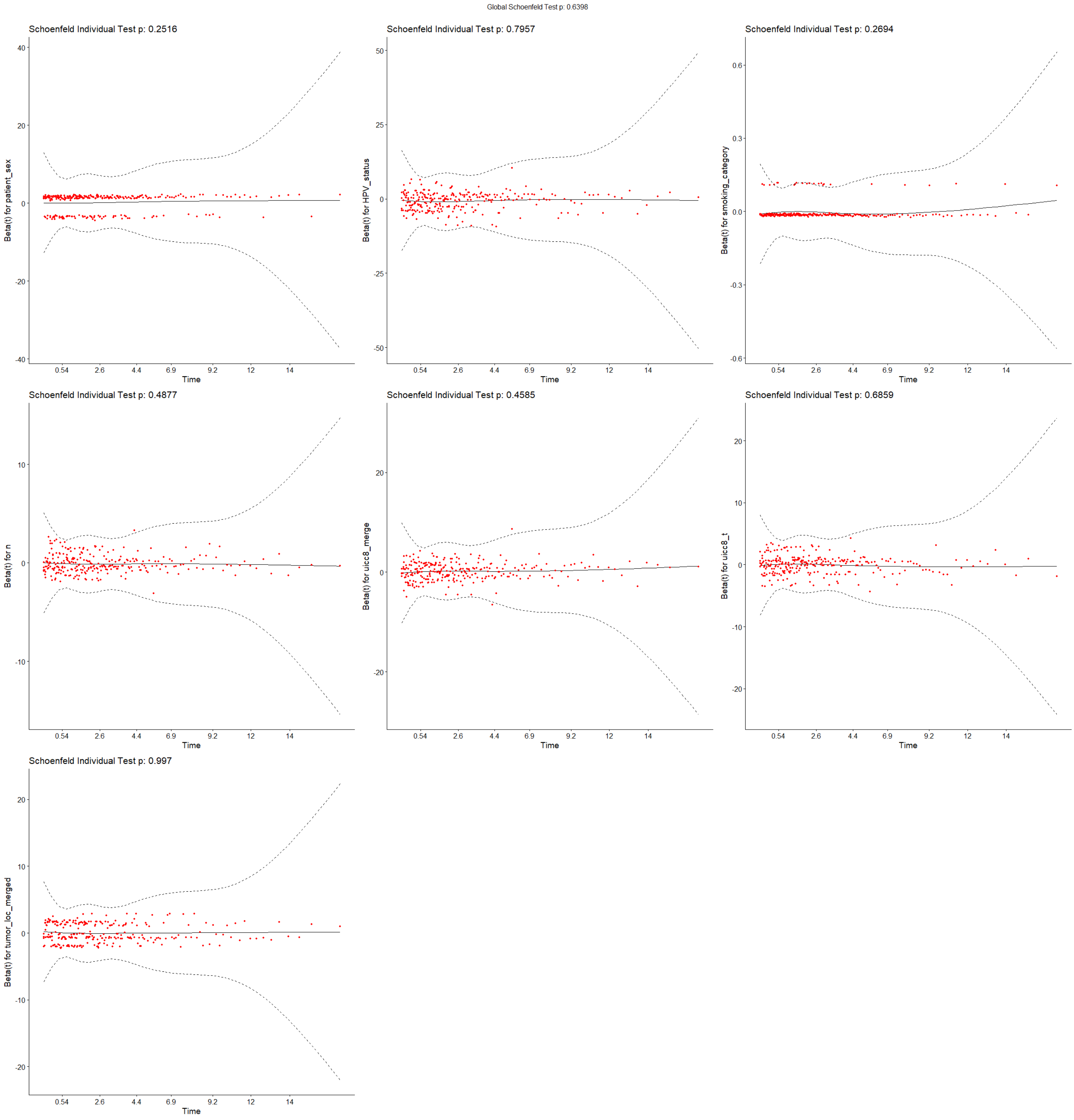

Figure S2. Proportionality of the variables tested by log-minus-log curves shown in Schoenfeld residual plots.
