# Supplementary figures and images for: The Incidence, Survival, and HPV Impact of Second Primary Cancer following Primary Oropharyngeal Squamous Cell Carcinoma: A 20-Year Retrospective and Population-Based Study
Source: Viruses. 2022 Dec 22;15(1):34. doi: 10.3390/v15010034 (PMC9867066; doi:10.3390/v15010034)

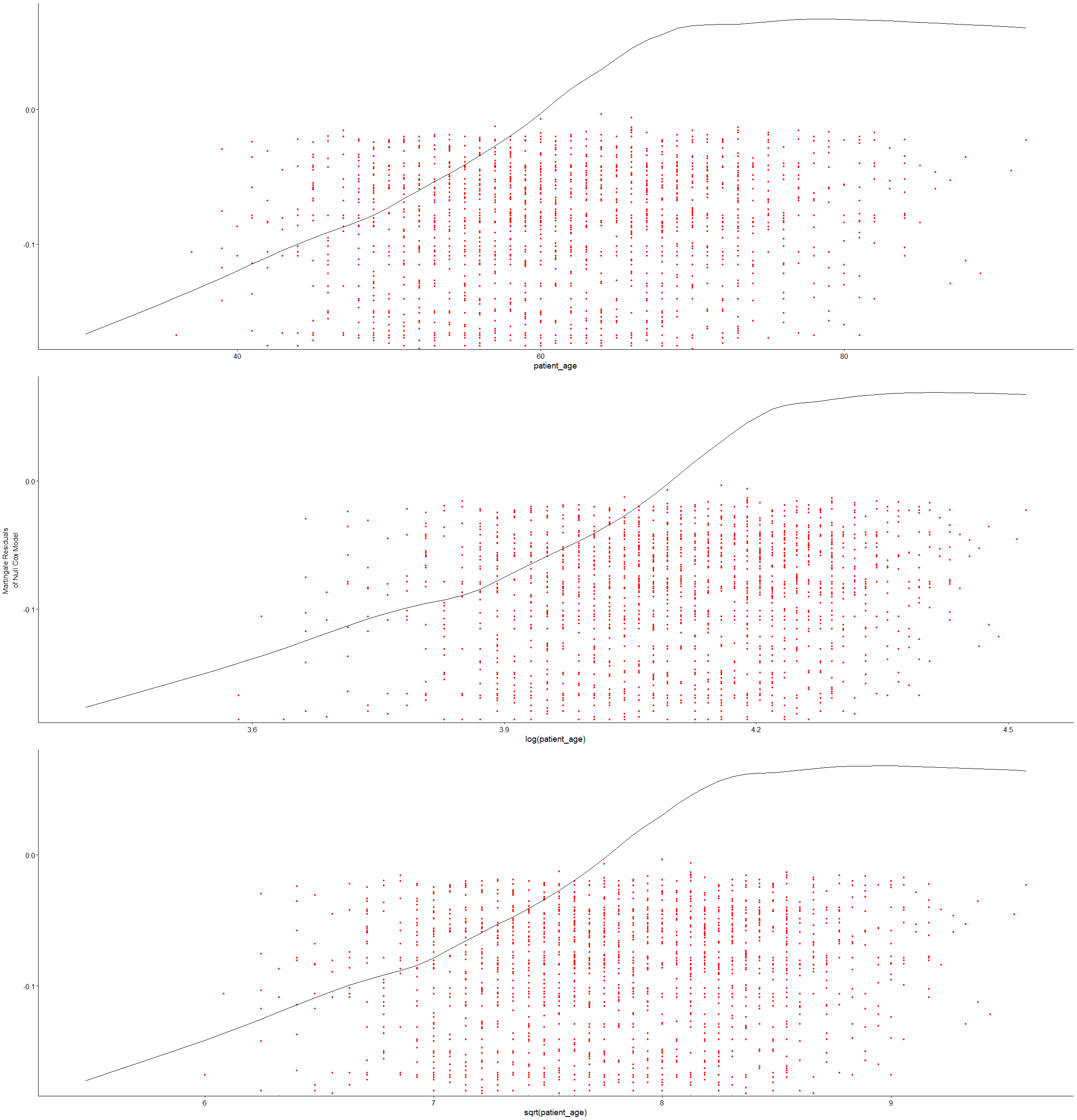

Supplement: Supplementary file 1 [file viruses-15-00034-s001.zip › Modelcontrol_S1_LA.png]

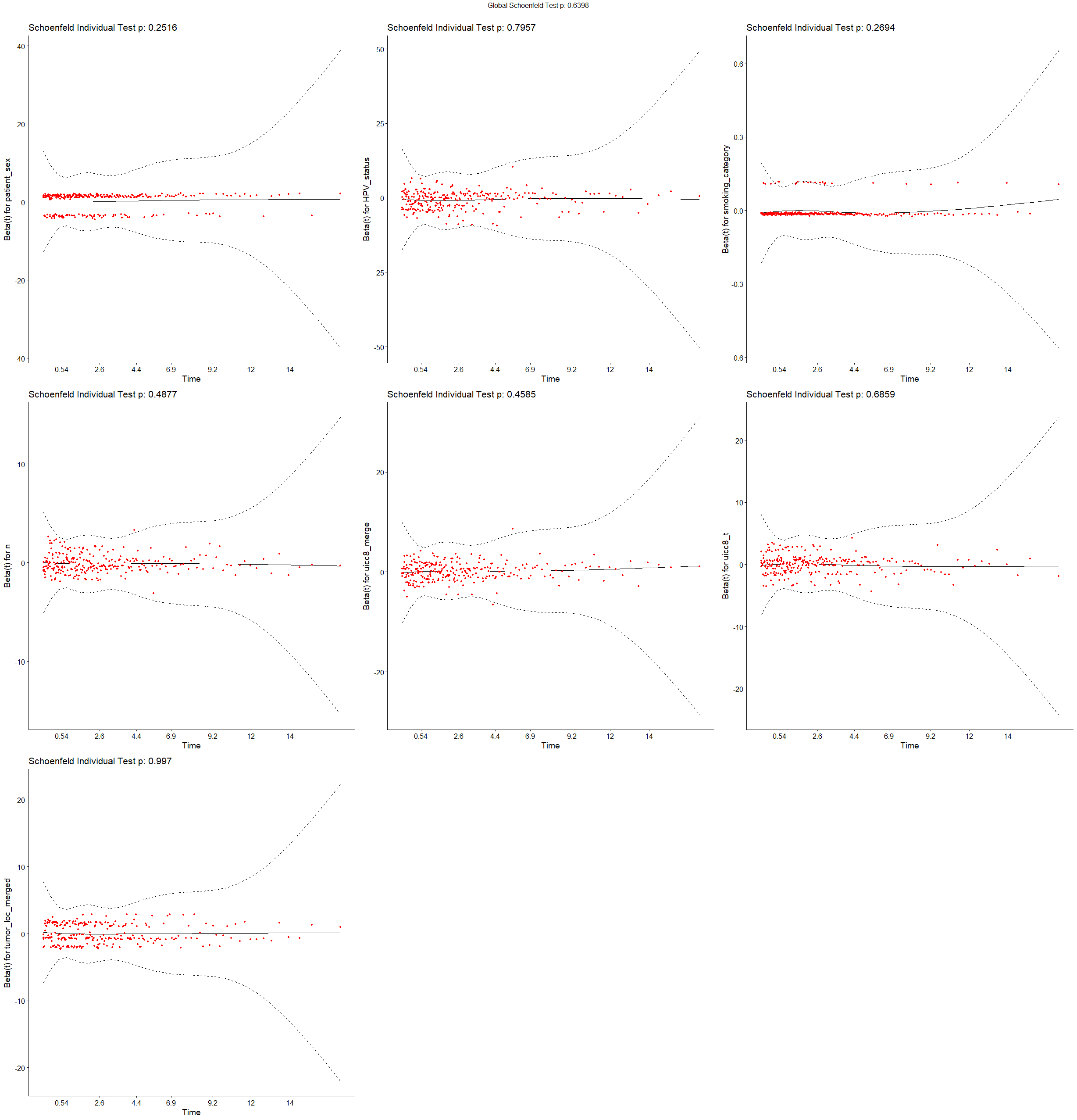

Supplement: Supplementary file 1 [file viruses-15-00034-s001.zip › Modelcontrol_S2_LA.png]
